# Teachers’ acute cardiovascular response to observed classroom disruptions and resulting chronic endocrine stress. Analysis of dynamic effects with global assessments for state space grids

**DOI:** 10.3389/fphys.2026.1880670

**Published:** 2026-09-16

**Authors:** Niklas Litzenberger, Gabriel Jenni, Alexander Wettstein

**Affiliations:** 1 Institute of Physics, Johannes Gutenberg-University, Mainz, Germany; 2 Institute for Research, Development and Evaluation, University of Teacher Education Bern, Bern, Switzerland

**Keywords:** chronic stress, classroom disruptions, dynamic systems, dynamic teaching analysis, observation, state space grids, time-series

## Abstract

**Introduction:**

Classroom disruptions are a major source of stress for teachers. However, teachers’acute physiological stress response to classroom disruptions and its consequences for chronic stress remain poorly understood.

**Methods:**

The present study assessed the acute cardiovascular stress response, i.e., heart rate (HR) and heart rate variability (HRV), and chronic stress, i.e., endocrine hormones in the hair. Trained raters coded observed classroom disruptions in 133 videotaped lessons of 36 teachers, during which an ECG measured their cardiovascular activity throughout the lessons. Through the novel Global assessments for State Space Grids method (GSSG), we examined teachers’ acute cardiovascular response to classroom disruptions.

**Results:**

Most teachers downregulated their HR and increased their HRV in reaction to classroom disruptions. Furthermore, three types of cardiovascular responses were identified: (1) maladaptive, (2) stable adaptive, and (3) unstable adaptive responses. Moreover, we introduced three measures of cardiovascular adaptiveness (i.e., HR regulation and HR short-term and long-term stability during classroom disruptions), which were linked to endocrine hormones in the hair longitudinally.

**Discussion:**

Results indicate that teachers with less adaptive cardiovascular responses exhibited a hormone pattern reflecting greater physiological dysregulation, whereas more adaptive responses were linked to more favorable hormone levels. The study shows that disruptions in social interactions affect acute cardiovascular responses and may influence long-term neuroendocrine hormone levels. Responding adaptively to social challenges may therefore be beneficial for both cardiovascular and neuroendocrine health. Moreover, the novel GSSG method proved useful for linking behavioral and physiological measures in social interactions.

## 1 Introduction

Exposure to a stressor can elicit an acute stress response in the autonomic nervous system (ANS). This physiological activation occurs rapidly, leading to an increase in the heart rate (HR) and a decrease in the heart rate variability (HRV). Behaviorally, an acute stress reaction can trigger a fight-or-flight response. However, such a physiological and behavioral response might not prove adaptive in some social environments. High cardiac vagal tone reflected in increased HRV can counteract a fight-or-flight response during the acute stress reaction (Thayer and Lane, 2000). The activation of the ventral vagus acts as a vagal brake, promoting relaxation and allowing us to respond adaptively even in socially challenging situations. This process may be particularly relevant for teachers while they are teaching. Teachers face numerous psychosocial stressors in their work, with classroom disruptions being a primary source of stress (Davis et al., 2020; Dicke et al., 2014; La Marca et al., 2023). However, teachers’ physiological stress reaction to classroom disruptions remains poorly understood.

Furthermore, frequent stressors combined with maladaptive responses or a lack of recovery can lead to our stress system becoming overactivated and dysregulated in the long term (Juster et al., 2010). Chronic stress can be measured in the neuroendocrine system, where stress is primarily reflected by the glucocorticoids cortisol and cortisone (Buoso et al., 2011; Staufenbiel et al., 2015). The antagonist to these stress hormones is dehydroepiandrosterone (DHEA), which buffers the effects of cortisol. The present study focuses on the effects of psychosocial stressors on the acute cardiovascular and long-term neuroendocrine stress reaction, considering adaptive and maladaptive responses. Specifically, a behavioral observation study was conducted to assess teachers’ acute (i.e., HR and HRV) and chronic stress (i.e., endocrine hormones in the hair) reactions and their cardiovascular adaptiveness (i.e., the extent of the ability to adapt) to observed classroom disruptions. Thereby, the study applied the novel Global assessments for State Space Grids (GSSG) (Litzenberger et al., 2025) method, enabling the examination of multidimensional stability metrics over time durations. GSSG can examine the interplay between physiological states (e.g., on the x-axis) and the respective social environments (e.g., on the y-axis) over time. Combining the x- and y-axis results in a holistic snapshot of the interaction system (i.e., classroom disruptions and HR or RMSSD). This novel method was developed to numerically capture the extent of moment-to-moment effects across multiple aspects of lessons using its own parameters. We adapted this method to examine the interplay between the classroom disruptions, which were rated using an indicator ranking system, and the teacher’s cardiovascular response, measured by HR and HRV.

## 2 Theoretical background

### 2.1 Classroom disruptions as social stressors

Teaching provides an ideal context for examining acute stress responses in social interactions, given its inherent unpredictability and high density of interactions. During the lesson, teachers face exceptionally dense and socially challenging interactions (Wettstein et al., 2024). Within this challenging interactional context, classroom disruptions constitute one of the most salient and demanding stressors. The present study defines classroom disruptions as interruptions in the teaching-learning process (Scherzinger and Wettstein, 2019). As such, classroom disruptions can emanate from students, the teacher, or external intrusions. Student disruption (e.g., agitation, cutting in, or threatening) hampers teaching effectiveness and decreases the opportunities for disruptive students and their classmates to learn. Teachers might further contribute to classroom disruptions by reacting inappropriately (e.g., shaming or ridiculing), thus aggravating an already problematic situation (Scherzinger and Wettstein, 2022, 2019). The term “classroom disruptions” does not refer to individual behavior, but to the reciprocal behavior of the participants in the interaction.

Because such reciprocal dynamics can repeatedly challenge teachers’ ability to maintain an orderly and supportive learning environment, classroom disruptions can negatively affect both students and teachers. They are a primary source of psychological and physiological stress in teachers (Boyle et al., 1995; Davis et al., 2020; Dicke et al., 2014; La Marca et al., 2023; McCormick and Barnett, 2011). For example, Davis et al. (2020) showed that challenging classroom behavior can elicit physiological stress responses in teachers, including changes in cardiovascular activity. From a psychological perspective, Dicke et al. (2014) showed that classroom disturbances played a mediating role in the association between teachers’ classroom management self-efficacy and emotional exhaustion. This is consistent with the ATLAS model of psychobiological teacher stress in classroom interactions (Wettstein et al., 2026), which conceptualizes teacher stress as an interplay between biological stress responses, psychological processes, and reciprocal classroom interactions. From this perspective, classroom disruptions are not only external stressors, but part of a dynamic interaction system in which teachers’ stress responses and classroom processes can mutually influence each other.

In the long run, stress negatively affects teachers’ work performance, mental and physical health, and social well-being, potentially resulting in burnout (Bellingrath et al., 2008; Evers et al., 2004; García-Carmona et al., 2019; Ingersoll, 2001). Consequently, teachers’ occupational problems and burnout diminish teaching quality, student motivation, and student performance (Blank and Shavit, 2016; Klusmann et al., 2016; Madigan and Kim, 2021; Shen et al., 2015). Given these far-reaching consequences, it is essential to understand teachers’ physiological and behavioral stress responses to classroom disruptions in order to prevent health problems among teachers.

### 2.2 The acute stress response as an adaptive reaction to challenges in the social environment

When teachers perceive classroom disruptions as threatening and lack sufficient resources to cope with them (Friedman-Krauss et al., 2014; Lazarus and Folkman, 1984; McCarthy et al., 2016), physiological, psychological, and behavioral stress responses may result (Lazarus, 1991; Mauss and Robinson, 2009). The acute stress response is executed by the ANS, which is comprised of two antagonistic components: The sympathetic nervous system (SNS) and the parasympathetic nervous system (PNS). When a perceived stressor threatens the organism’s homeostasis, the SNS is activated, and the catecholamine hormones adrenaline and noradrenaline are released into the bloodstream. SNS activation occurs rapidly and may include triggering a fight-or-flight response. The PNS enables the restoration of homeostasis (e.g., maintaining a balanced body temperature or blood pressure) through delayed responses, and it predominates during quiet, resting activities.

ANS activation can be measured with the HR and HRV. While HR corresponds to the number of contractions (beats) per minute (Pinel, 2010), HRV refers to the beat-to-beat alterations between successive heartbeats (Task Force of the European Society of Cardiology and the North American Society of Pacing and Electrophysiology, 1996). A measure for HRV often used in research is RMSSD (the root mean square of distances between successive R peaks, so-called interbeat intervals; Shaffer et al., 2014). It captures the beat-to-beat variance in heart rate and is considered the standard measure of vagally mediated parasympathetic cardiac control. SNS activation results in an increase of HR and a decrease of HRV (Task Force of the European Society of Cardiology and the North American Society of Pacing and Electrophysiology, 1996). From the perspective of the neurovisceral integration model, elevated HRV indicates greater ability to regulate stress and arousal (Thayer and Lane, 2000; Thayer et al., 2009) and reflects a good self-regulatory capacity (e.g., emotion regulation) and adaptability (i.e., the ability to adapt) to stressors (McGee et al., 2023).

It can be assumed that in acute reaction to classroom disruptions, HR increases and HRV decreases (Davis et al., 2020). Previous research has shown that work stress is associated with lower HRV, suggesting that occupational stressors may impair autonomic regulation by reducing parasympathetic, vagally mediated cardiac control (Chandola et al., 2010; Jarczok et al., 2013; Järvelin-Pasanen et al., 2018). However, few studies examined this relationship in the context of concrete stressful situations in teachers’ everyday lives (Beuchel and Cramer, 2023). Furthermore, studies have failed to find an association between stressful events and changes in teachers’ HR or HRV (Pieper et al., 2010). This result concurs with a recent review indicating overall inconsistent findings on the relationship between occupational stress and cardiovascular parameters in teachers (McGee et al., 2023). Still, specific studies found that teachers have a higher HR and lower HRV on workdays compared to leisure days (Kühne et al., 2024; Schwerdtfeger et al., 2008; Wettstein et al., 2020). This finding suggests that specific recurring stressors, such as classroom disruptions, may trigger physiological stress responses via the SNS in teachers on their workdays.

### 2.3 Adaptive reactions to social stressors: the vagal brake

Phylogenetic patterns, such as the fight-or-flight reaction, have evolved as quick responses to danger. Rapid autonomic mobilization of physiological resources enhances the organism’s chances of surviving immediate threats by preparing the body for swift action. However, this mobilization triggered by a fightor-flight reaction may be inadequate for managing social challenges in the classroom as it impairs social functioning (von Dawans et al., 2021; Porges, 1995). In such situations, stronger parasympathetic vagal control, as reflected in higher HRV, may counteract excessive mobilization. Because higher cardiac vagal tone is associated with stronger inhibitory control and self-regulatory capacity, it may also promote greater behavioral flexibility and adaptability (Thayer and Lane, 2000; Thayer and Koenig, 2025). The myelination of the ventral vagus allows rapid transmission of nerve signals, enabling physiological downregulation through highly dynamic adaptation to environmental changes. The ventral vagus can get activated when an individual feels safe and tries to overcome a social challenge through communicating with others or interpreting their social signals (Porges, 2003). As several studies demonstrate, the ventral vagus can act as a vagal brake, regulating the HR and inhibiting the sympathetic-adrenal system from influencing the heart (Shaffer and Ginsberg, 2017; Thayer et al., 2009; Uijtdehaage and Thayer, 2000). Thus, physiological downregulation, characterized by decreased HR and increased HRV, may help teachers avert mobilization during challenging social interactions. It would support maintaining composure and more effectively navigating socially challenging situations. In contrast, physiological upregulation is characterized by increased HR and decreased HRV and is exhibited during a fight-or-flight reaction.

Complementing this vagal-brake perspective, the neurovisceral integration model provides a broader framework for understanding HRV as an index of adaptive self-regulation (Thayer and Lane, 2000; Thayer et al., 2009). According to this model, HRV reflects the capacity of the central autonomic network to flexibly regulate physiological arousal in response to changing environmental demands.

It may be adaptive for teachers to adjust and vary their response to the respective environment (Mischel, 2013). In a metaphorical sense, teachers are like drivers of a car. They can adapt their physiological and behavioral responses to the terrain (i.e., the respective social situation). When a hill appears (i.e., classroom disruptions), gears need to be switched (down- or upregulation of HR and HRV). While adaptive drivers downshift at the right moment to climb a hill (i.e., HR downregulation due to greater vagal tone in response to classroom disruptions), others upshift, making it harder to ascend (i.e., HR upregulation or sustained cardiovascular activation in response to classroom disruptions). Once the correct gear is engaged, the hill will have both steep and less steep sections during the climb (i.e., changes in classroom disruption levels). Steadily finding the clutch’s friction point and, if necessary, switching gears again is essential and demonstrates adaptive driving. Compared to maladaptive driving, adaptive driving leads to lower engine costs (i.e., psychobiological health of teachers).

After down- or upregulation in response to the initial classroom disruption, an adaptive response is likely reflected in HR and HRV remaining stable at this new physiological state despite high levels of classroom disruptions. Ultimately, the adaptiveness of a physiological system emerges in relation to its respective environment. An adaptive physiological system recognizes changes in its environment, can adequately adjust to these new environmental conditions, and remains stable in the new state, preserving homeostasis. In contrast, a maladaptive physiological system may shift into a new state characterized by instability, absence of homeostasis, and dysregulation. It can be assumed that adaptive regulation of short-term cardiovascular processes also has a long-term beneficial effect on teacher health (Hughes et al., 2018; Madigan and Kim, 2021).

### 2.4 When stress becomes chronic

The acute stress response prepares the body for the psychosocial challenges in the classroom and is not harmful in the short term (Birbaumer, 2010). However, chronic activation of physiological systems responding to stressors can harm the body. Chronic stress endangers teachers’ health by potentially leading to long-term physiological stress consequences (e.g., altered ANS activity, hypothalamic-pituitary-adrenal axis dysregulation, subclinical inflammation) (Juster et al., 2010). In the present study, while examining the ANS in relation to the acute stress response, we focus on the neuroendocrine system in the context of chronic stress.

Following the rapid activation of the ANS in response to acute stress, the hypothalamic-pituitary-adrenal (HPA) axis is engaged as a slower, subsequent neuroendocrine stress system. Its activation leads to the secretion of cortisol into the bloodstream, which is subsequently converted into inactive cortisone (Staufenbiel et al., 2015). The concentration of cortisol (HCC) and cortisone (HCO) in the hair reflects long-term cumulative cortisol and cortisone levels, respectively (Russell et al., 2012). Thus, chronic stress can be measured with HCC and HCO. Reviews and meta-analyses have shown that individuals exposed to chronic stress (e.g., due to unemployment, shift work, caregiving, or natural disasters) usually exhibit higher levels of HCC compared to control groups (Staufenbiel et al., 2013; Stalder et al., 2017). To date, HCO has received comparatively less research attention. Findings have shown positive associations between HCO and stressful events, mental and physical stress, as well as subjective stress perception (Ullmann et al., 2016; Vanaelst et al., 2013). Other studies, however, have found no relationship with perceived stress (Davison et al., 2019; Musana et al., 2020). Nevertheless, the combination of HCC and HCO may represent a more robust stress hormone index and provide more comprehensive results than HCC alone (La Marca et al., 2024; Penz et al., 2018).

Additionally, the present study investigated DHEA. DHEA is an anabolic steroid, a precursor of the sex steroids androgen and estrogen, and an antagonist against the effects of cortisol (Buoso et al., 2011; Pinto et al., 2015). It plays a central regenerative role in modulating the physiological regulatory systems involved in the stress response, particularly the HPA-axis (Maninger et al., 2009). Similar to cortisol, DHEA levels rise in reaction to stressful events (Kamin and Kertes, 2017; Maninger et al., 2009). Lower DHEA levels may heighten neural vulnerability to cortisol’s neurotoxic effects (Alhaj et al., 2006; Suzuki et al., 2004). In the occupational context, employees’ self-reported well-being has been positively linked to hair DHEA levels (Girardi et al., 2024). Therefore, DHEA is also considered a marker of psychophysiological resilience and well-being (Russell et al., 2012; Rutkowski et al., 2014).

Chronic stress can promote physiological dysregulation. Physiological dysregulation occurs when physiological systems shift from normal homeostatic functions (e.g., body temperature, oxygen supply) into abnormal ranges due to the extended release of stress hormones (Juster et al., 2010). Consequently, this dysregulation results in a maladaptation of interdependent systems and can be damaging to our health (McEwen, 1993). Dysregulation can manifest in asymmetry between physiological systems (e.g., the SNS and HPA-axis) (Schneider et al., 2025) but also within a single system. Within the HPA-axis, HCC and DHEA have been previously studied regarding physiological dysregulation (Dutheil et al., 2021; Russell et al., 2012; Rutkowski et al., 2014). The ratio between the two hormones HCC and DHEA allows for the examination of their opposing effects simultaneously (Sollberger and Ehlert, 2016). Considering the two hormones in relation to each other captures the preferential production of one hormone over the other. Thus, the C/DHEA ratio is considered a measure of catabolic/anabolic balance, with higher levels or an increase over time being associated with a higher susceptibility to physiological dysregulation (Epel et al., 2018; Wolkowitz et al., 2001).

In sum, classroom disruptions can trigger acute stress responses in teachers, characterized by immediate cardiovascular activation and the subsequent engagement of the HPA-axis. When acute stress responses are frequent or prolonged, they may lead to chronic stress, which the dysregulation of the HPA-axis reflects. This process, in turn, might impair cardiovascular function and reshape acute cardiovascular reactivity.

### 2.5 Present study and research questions

This study is part of a larger ambulatory assessment project on stressful classroom interactions (Wettstein et al., 2021). An ambulatory assessment enables the capture of an individual’s real-life working situations. Thereby, it enhances ecological validity and our understanding of the complex interplay between psychosocial and physiological factors. Existing research on psychosocial stressors (i.e., classroom disruptions) is mainly based on self-reports that assess subjective perceptions (Aloe et al., 2014b; Liu et al., 2017). Studies based on questionnaires for measuring teachers’ subjective stress experiences are primarily cross-sectional and rarely consider long-term physiological stress reactions (Aloe et al., 2014b; Hughes et al., 2018; McGee et al., 2023). However, for a comprehensive understanding of teachers’ work stress, it is essential to assess not only psychological but also physiological processes and consequences. The present study aims to investigate the effects of objectifiable psychosocial stressors in real-life work experiences on teachers’ cardiovascular responses by analyzing dynamic patterns using Global assessments for State Space Grids (GSSG). In particular:

1. In response to classroom disruptions, how many teachers show an HR and HRV (i.e., RMSSD) downregulation or upregulation, and how stable is this regulation?
2. Can more or less adaptive HR and HRV (i.e., RMSSD) response types to observed classroom disruptions be identified?
3. To what extent do teachers’ HR and HRV (i.e., RMSSD) increase, decrease, or remain stable in response to observed classroom disruptions?
  Moreover, the study exploratorily examines the link between teachers’ acute cardiovascular responses and endocrine markers of chronic physiological stress and physiological dysregulation longitudinally. Specifically:
4. How is cardiovascular adaptiveness associated with the endocrine hormones HCC, HCO, DHEA, and the C/DHEA ratio?

## 3 Methods

### 3.1 Participants

The sample included 36 teachers (25 females, age of 39.14 ± 12.04 years) with 162 ± 43 minutes of videotaped lessons per teacher. Participants were recruited via flyers and circular emails. Inclusion criteria for participation in the study were employment as a primary or secondary teacher in the canton of Bern and a workload of at least 16 lessons per week (equivalent to at least 60% employment). Exclusion criteria were working outside of the canton of Bern, acute infections, cardiovascular or other chronic diseases, usage of cardiovascular drugs or other medication in the past two months (except phytopharmaceuticals), substance abuse, consumption of psychoactive substances in the last four weeks, more than two standard alcoholic drinks per day, smoking more than ten cigarettes per day, long-distance flights within the last two weeks, as well as pregnancy. All teachers were screened for inclusion and exclusion criteria in a short interview. Enrolled participants signed informed consent. The study was approved by the ethics committee of the canton of Bern and by the Internal Review Board (IRB) of the University of Bern. It was conducted in strict compliance with current data protection laws and in accordance with the Declaration of Helsinki.

### 3.2 Design and procedure

The design and procedure of the present study are based on a psychobiological approach to stressful interactions in the classroom as proposed by Wettstein et al. (2021). Thus, this study followed a longitudinal psychobiological ambulatory assessment that relies on an ecological approach to study the participants in their “natural habitats” (Trull and Ebner-Priemer, 2013). An ambulatory assessment allows for capturing teachers’ real-life working situations. Thereby, it enhances ecological validity and our understanding of the complex interplay between psychological, physiological, and social factors. The study included two measurement points (baseline *t*_0_ and one-year follow-up *t*_1_). Combining electrophysiological measures, observation, and endocrine hormones represents a multimodal approach and reduces the common method bias. **Figure 1** illustrates the timeline of the design and procedure.

**Figure 1:**
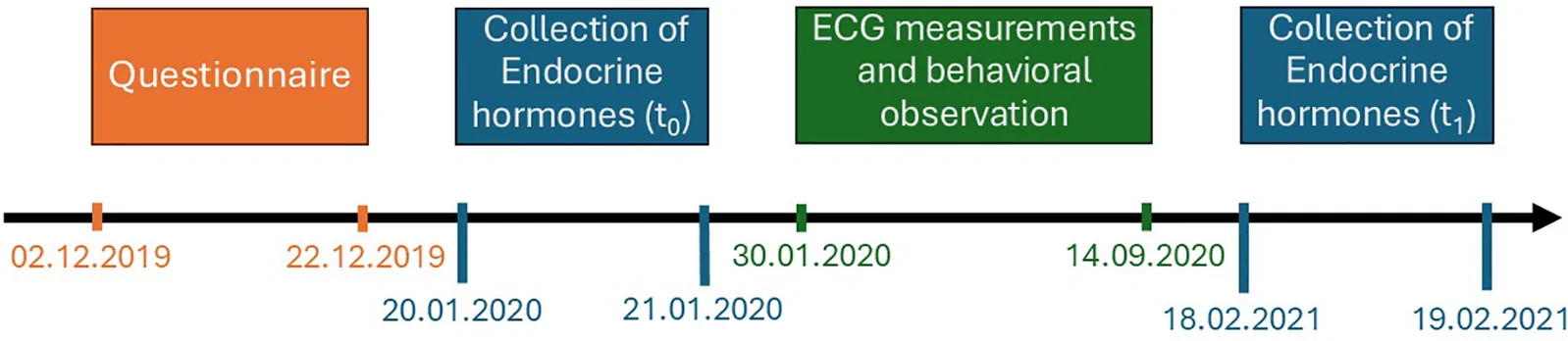
Timeline of the design and procedure.

At the first measurement (*t*_0_), teachers provided information on demographic variables (e.g., sex, age, years of teaching experience). The electrocardiogram (ECG) measuring HR and HRV was recorded during the observed lessons. Hair samples measuring endocrine hormones (i.e., cortisol, cortisone, and DHEA) were collected 120.34 ± 84.39 days after completing the questionnaires, and 67.63 ± 18.40 days prior to classroom observations and ECG. One year later (*t*_1_), endocrine hormones were reassessed.

### 3.3 Measures

#### 3.3.1 Electrophysiological measures

To adequately account for methodological aspects in HRV measures (Laborde et al., 2017), we followed the guidelines of the Task Force of the European Society of Cardiology and the North American Society of Pacing and Electrophysiology (1996). We recorded the ECG using the EcgMove4 sensor from Movisens, Version 1.9.31.0 (movisens GmbH, Karlsruhe, Germany), which features adhesive electrodes that are easy to apply. It records a single-channel ECG at a sampling rate of 1,024 Hz. The sensor was placed below the left breast in all participants using adhesive electrodes, and ECG data were recorded continuously during the lessons.

The processing and analysis of the ECG data were conducted using Kubios HRV Premium version 3.5.0 (Tarvainen et al., 2014). Since artifacts can affect the reliability of HRV measurements (Tarvainen et al., 2014), all ECG data were carefully checked and corrected in Kubios. First, the ECG signal was visually inspected together with the RR-interval series. Second, the automatically detected R-peaks were checked manually. If Kubios missed a beat, detected an additional beat, or placed an R-peak marker incorrectly, this was corrected by hand. Third, segments with noise, movement artifacts, baseline shifts, or ectopic beats were inspected in detail. Segments were retained when the R-peaks were clearly visible and the RR-interval series was not distorted. If the R-peaks were affected, they were manually corrected. After this manual correction, Kubios’ automatic correction was used only to correct remaining clear artifacts. The percentage of corrected beats for each segment did not exceed 5%.

#### 3.3.2 Behavioral observation

GoPro cameras and microphones recorded four 45-minute lessons (following a three-day acclimation period to minimize potential reactivity). The video material was coded and analyzed by four trained external observers in an event-sampling procedure using the BASYS observation system (Wettstein, 2008) with MAXQDA 2020 (VERBI Software, 2019).

The BASYS observation system allows for coding of disruptive behavior in school settings. Observed classroom disruptions included a working atmosphere marked by interruptions, lack of concentration, and restlessness. Before coding, all four observers were trained to a criterion of .80 (Cohen’s kappa), and unclear episodes were discussed regularly. 11% of the coded video material was recoded, resulting in an inter-rater reliability of a Cohen’s kappa of .85. The level of observed classroom disruptions was determined continuously on a scale of one to four, with one indicating “hardly any disruptions” and four indicating “teaching hardly possible any more”.

#### 3.3.3 Endocrine markers

Hair strands were cut from the posterior vertex as close to the scalp as possible. Hair cortisol, cortisone, and DHEA concentrations were determined from the 3 cm segment closest to the scalp. Given an average hair growth rate of 1 cm per month, this segment represents the cumulative glucocorticoid (GC) secretion over a three-month period preceding sampling. The washing procedure and GC extraction followed the laboratory protocol described by Gao et al. (2013). All samples were analysed by liquid chromatography coupled with tandem mass spectrometry (LC-MS/MS). This analysis’s lower limits of quantification (LOQ) are below 0.1 pg/mg for cortisol, cortisone, and DHEA. The inter- and intra-assay coefficients of variance for cortisol, cortisone, and DHEA are below 15% (Gao et al., 2013). HCC, HCO, and DHEA were reassessed one year later.

Due to non-normal distribution, age and all endocrine hormones were log-transformed. In addition to examining HCC and HCO separately, the sum of the two was calculated (i.e., HCCHCOsum; La Marca et al., 2024; Penz et al., 2018). The C/DHEA ratio was calculated by dividing the HCC values by the DHEA values and then log-transforming the resulting values (Sollberger and Ehlert, 2016). Three participants had insufficient hair length to collect data from. One year later, we had a drop-out of an additional three participants. Furthermore, one participant was excluded from all calculations with HCC, HCO, HCCHCOsum, DHEA, and C/DHEA ratio due to the use of a hair product containing cortisone. Correlations between endocrine hormones and the cardiovascular variables were always controlled for age, sex, body mass index, and absence of the teacher during the lesson (i.e., the percentage of time the teacher was outside the classroom during the four lessons). Cortisol levels rise with ageing and may differ by sex (Feller et al., 2014; Roelfsema et al., 2017). DHEA levels tend to decline with ageing (Maninger et al., 2009) and DHEA acts as a metabolic intermediate in the production of sex hormones (Labrie, 2010). Absence of the teacher during the lesson was controlled for, as physical activity during the absence could have affected the ECG data.

#### 3.3.4 Time series

The ECG and behavioral observation data from the lessons were divided into one-minute segments (standard in short-term cardiovascular parameters; Laborde et al., 2017). The segments of the ECG data were created in Kubios and those of the observation data in MAXQDA. The two time series (ECG and behavioral observation) were then temporally aligned in R (Version 4.4.1) and merged into a single timeseries data frame for each teacher. Segments during which the teacher was absent from the classroom or segments during the break between lessons were removed. The time series of three teachers contained missing segments for HR and RMSSD. In one of these three time series, 11.64% of the segments were imputed, while in the other two, 4.42% and 1.89% were imputed, respectively. Missing segments were imputed by calculating the mean of the HR and RMSSD values from the preceding and following segments. The number of missing segments never exceeded four in a row. In one time series, the first seven segments were missing, but the time series could still be successfully included in our analysis. Since we obtained 162 ± 43 segments per teacher on average, these few missing segments were a negligible interference for our data.

The range for a teacher’s HR and RMSSD is individual (Pinel, 2010). Thus, it is not meaningful to compare the total change in the HR or RMSSD between individual teachers, because a high HR for one teacher could be a low HR for another teacher. Instead, comparing the changes in HR and RMSSD within the total range of each teacher is more meaningful (Pinel, 2010). This way, a HR of 100 bpm of a teacher with a range of 70 to 110 bpm is comparable to a HR of 80 bpm of another teacher with a range between 60 and 80 bpm. Hence, we identify the range of HR and RMSSD for each teacher and categorize the mean value of HR for each minute into five equally sized segments. This approach leads us to a value between 1 and 5 to measure and compare the time series of each teacher’s HR and RMSSD. We also tested a higher number of segments with similar results. However, since the five segments provide visually more apparent differences for different time sections with the GSSG method, we will present the results based on a five-segment scheme.

### 3.4 Data analysis with global assessments for state space grids

To analyze whether a teacher downregulated or upregulated HR and RMSSD, we need to examine the time series of the cardiovascular response to classroom disruptions. Furthermore, we need to distinguish between two states in the time series: before and during the classroom disruptions. Theoretically, there could also be a third state after the classroom disruptions decrease again. However, in no observed lesson did the observed classroom disruptions reduce to an extent similar to the first state, indicating a third state with only a few disruptions. Thus, we did not need to take a third state into account and will only examine the temporal development of the states before and during classroom disruptions.

A method for analyzing the temporal development of two aspects is the State Space Grids method (SSG) (Hollenstein, 2013). Although the SSG method is well-established for analyzing teacher-student interactions (Mainhard et al., 2012; Pennings et al., 2018; Pennings and Hollenstein, 2020), the present study applies it for the first time to teachers’ cardiovascular measures. SSG mainly focuses on the analysis of a specific combination of values in the x- and y-axis (Hollenstein, 2013; Turner and Christensen, 2020). For example, it can determine how long and at what frequency a specific HR value and observed classroom disruptions occur during a lesson.

However, to answer research question one, we need to analyze more “global” trends, such as the overall changes in HR and RMSSD before and during classroom disruptions. Furthermore, for research question three, we need to examine and compare the extent to which the time development of the HR and RMSSD measurements remains similar across states to analyze how stable the teachers’ regulation is. Since SSG primarily focuses on the analysis of specific temporal combinations, it is not well-suited for analyzing global trends. However, the authors (Litzenberger et al., 2025) developed a novel extension to SSG to analyze global trends with Global assessments for State Space Grids (GSSG), enabling the examination of multidimensional stability metrics over time durations.

This novel method allows the analysis of changes in HR and RMSSD due to classroom disruption. It aims to gain metrics of multidimensional stabilities over time durations and visualizes them, using parameters for the pattern and stability of whole-time states. These GSSG parameters enable the comparison of two stability types: minute-to-minute (short-term) and overall (long-term) stability of HR and RMSSD across different states during the lesson. This way, it is possible to numerically compare the cardiovascular response in the lesson states before and during classroom disruptions.

To illustrate the way we use the GSSG method, we will examine an example of the HR and the classroom disruptions of two specific teachers, visualized with the GSSG method in **Figures 2a, b**. The *x*-axis shows the teacher’s HR at each minute, and the *y*-axis shows the corresponding observed classroom disruptions. The analysis of RMSSD is very similar to that of HR, which is why we will only examine an example of HR changes. Since we have five sections for HR and classroom disruptions, we have five possibilities on the x- and y-axes, resulting in a 5x5 grid. The blue points represent these two dimensions during the first state, when only a few classroom disruptions occur. In contrast, the purple points represent the second state, where classroom disruptions occur almost always. **Figure 2a** shows one example of this lesson state comparison, but we can find these two distinct states (before and during classroom disruptions) for all teachers.

**Figure 2:**
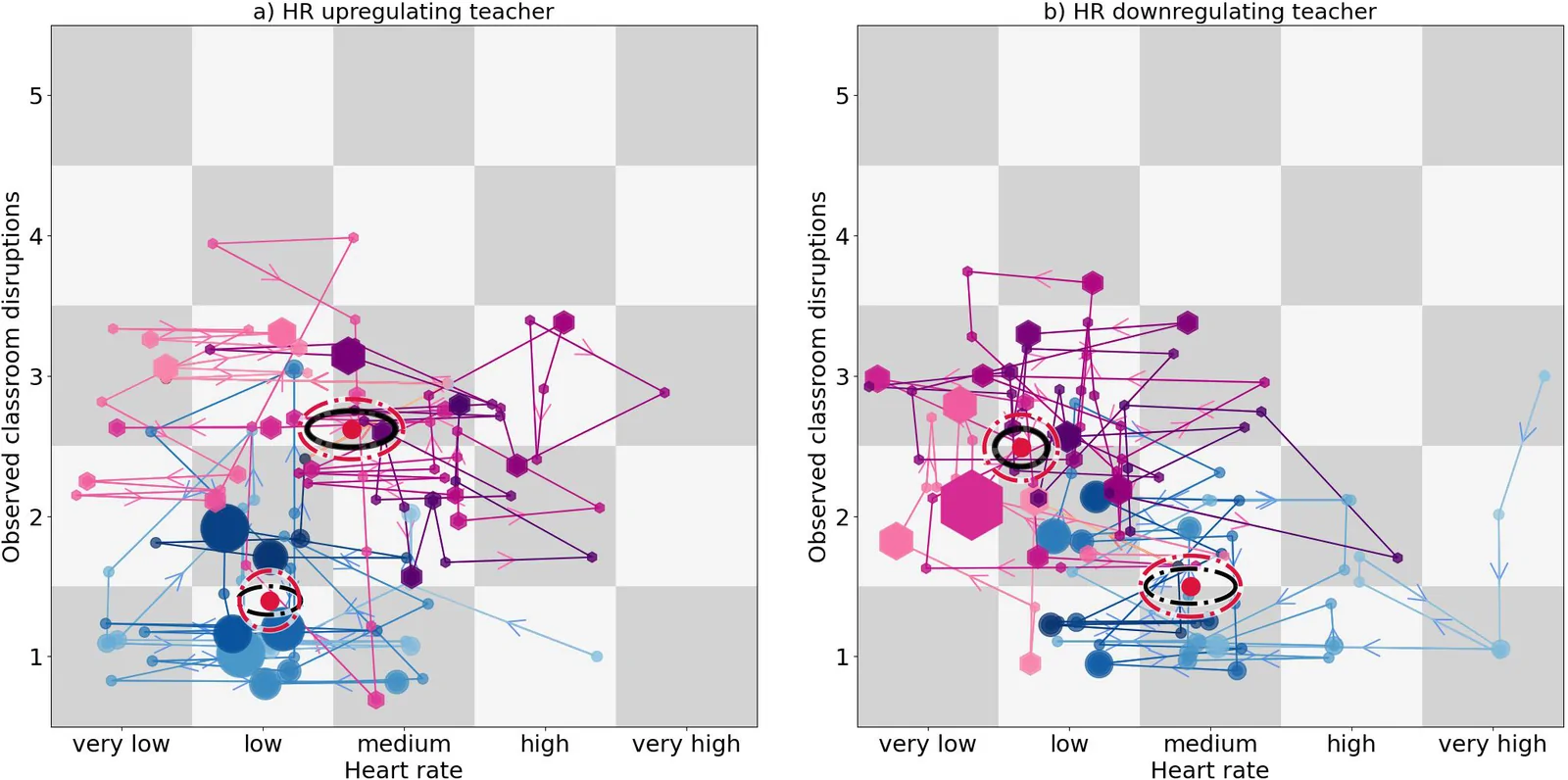
GSSG with a HR **(a)** upregulating and **(b)** downregulating teacher during classroom disruptions.

In the case of **Figure 2a**, we can see that in the second state, the HR increases, because the purple ratings have higher values on the x-axis. Thus, this teacher upregulated the HR. In contrast, **Figure 2b** shows a teacher downregulating the HR, as indicated by the lower purple ratings on the x-axis. Through this clear visual indication, we can determine whether a teacher upregulates or downregulates to answer research question one.

However, through this visual indication of cardiovascular up- and downregulation alone, we can not assess the extent of the regulation to answer research question three. GSSG provides a parameter for the extent of state changes by determining the center of each state. The center of each state tells us the overall value of classroom disruptions and HR. The visual representation of the state’s center is the red point at the center of both ellipses. Comparing the state center of the first state to that of the second state provides a measure of the extent of the teacher’s up- or downregulation. In case of **Figure 2a**, we can find an increase for the HR from 2.05 to 2.63 and for the classroom disruptions from 1.40 to 2.62. Thus, we can also calculate the overall change in HR, which is the difference between both states/red points.

We call this overall change in the teacher’s HR $\text{Δ}\bar{HR}$ and calculate it through the following formula:

(1) $$\text{Δ}\bar{HR}=\frac{1}{t_{1}}\sum_{t=0}^{t_{1}}HR(t)-\frac{1}{t_{2}-(t_{1}+1)}\sum_{t=t_{1}+1}^{t_{2}}HR(t),$$

where *t*_1_ is the last time step of the first state (before disruptions), *t*_2_ is the last time step of the second state (during disruptions), and *HR*(*t*) is the HR of the time step *t*. Through the formula’s first sum, we calculate the average HR in the first state and the average HR of the second state in the second sum. Thus, throughout this study, we will use the sign convention of $\text{Δ}\bar{HR}>0$ if the teacher downregulates and $\text{Δ}\bar{HR}<0$ if the teacher upregulates. For the example in **Figure 2a**, the formula results in $\text{Δ}\bar{HR}=-0.58$, as the teacher upregulates the HR. In contrast, in **Figure 2b**, we find a decrease in the overall change in HR from 2.86 to 1.66. Thus, the teacher downregulates the HR, and the formula returns a positive value for $\text{Δ}\bar{HR}$ of 1.20.

Using the state center comparison, we can measure the extent of up- or downregulation, but not the extent to which this regulation is stable. Looking at **Figure 2a**, the purple points spread out more in the grid than the blue points, indicating that the HR is less stable. The GSSG method provides two parameters to measure variability, which we will transform into measures for stability through a simple formula. We call these two types short-term and long-term stability.

The GSSG method can also visualize the short-term and long-term stability through the ellipses within each state. The red ellipse visualizes the long-term stability for each dimension, and the black ellipse visualizes the short-term stability. A small ellipse indicates a higher stability, while a more spread-out ellipse indicates less stability. Through this visualization, we can see that the long-term and the short-term stability are lower for the second state in **Figure 2a**, because both ellipses are larger for the second state. In contrast, **Figure 2b** shows an increase in long-term and short-term stability. This way, we can see whether the teacher up- or downregulates stably. However, we also want to measure the extent of this stability.

We measure the first type of stability through transforming the GSSG’s parameter for minute-to-minute variability, Mean Travel Distance (MTD) (Litzenberger et al., 2025). For example, the MTD of 0.404 in the second state of **Figure 2b** indicates that from one minute to the next, the values change by 0.404 on average. Higher values of the MTD indicate more minute-to-minute change on average. However, since we want to measure stability rather than variability, we should use a metric where higher values indicate greater stability. This is why we transformed this measure of variability into a measure of stability, which we call short-term stability (STS), as it indicates stability over short time scales from minute to minute. We gain the STS, using the following formula: *STS* = 1 − *MTD*. To examine changes in stability, we calculate the difference in STS between the first and second states. In case of **Figure 2**, we find a decrease in STS for the upregulating teacher (Δ*STS* = −0.107) and an increase in STS for the downregulating teacher (Δ*STS* = 0.114).

Similar to the STS, we calculated the second type of stability, which indicates the overall spread on both the *y*- and *x*-axes, referred to as the long-term stability (LTS), based on the GSSG method’s Mean Standard Deviation (Litzenberger et al., 2025). This type of stability, measured by *LTS* = 1 − *MSD*, indicates how stable HR and classroom disruptions are over the entire duration of each state. In case of **Figure 2a**, we find a LTS of 0.735 in the first state and a LTS of 0.636, which gives us a decrease of Δ*LTS* = −0.099. In contrast, **Figure 2b** has a Δ*LTS* of 0.047, indicating also an increase in long-term stability.

In summary, we obtain three measurements of a teacher’s cardiovascular adaptiveness in response to classroom disruptions using this GSSG approach: the overall change in HR ( $\text{Δ}\bar{HR}$), as well as changes in short-term stability ( ΔSTS) and long-term stability ( ΔLTS). We computed these three cardiovascular adaptiveness measures by calculating the difference between the first and second states. While positive values in $\text{Δ}\bar{HR}$ represent HR downregulation, negative values represent HR upregulation. Positive values in the difference variables ΔSTS and ΔLTS indicate an increase in HR stability from the first to the second state. In contrast, negative values indicate a decrease in HR stability.

As with HR, we also examine RMSSD by combining classroom disruptions and RMSSD. This way, we also gain $\text{Δ}\bar{RMSSD}$, ΔSTS, and ΔLTS for this combination. We calculate $\text{Δ}\bar{RMSSD}$ in a similar way as ΔHR in Equation 1. However, the sign convention for $\text{Δ}\bar{RMSSD}$ is different: $\text{Δ}\bar{RMSSD}$ is positive if the RMSSD of the teacher increases and $\text{Δ}\bar{RMSSD}$ is negative if the RMSSD of the teacher decreases. Due to the difference in sign convention, the formula for $\text{Δ}\bar{RMSSD}$ shifts the position of the first and the second sum of Equation 1. This shift leads to Equation 2:

(2) $$\text{Δ}\bar{RMSSD}=\frac{1}{t_{2}-(t_{1}+1)}\sum_{t=t_{1}+1}^{t_{2}}RMSSD(t)-\frac{1}{t_{1}}\sum_{t=0}^{t_{1}}RMSSD(t),$$

where, again, *t*_1_ and *t*_2_ are the last time steps of the first and the second state, and *RMSSD*(*t*) is the teacher’s RMSSD at time step *t*.

To clearly distinguish between the STS and LTS corresponding to HR and RMSSD, we use the notation of Δ*LTS_HR_* and Δ*STS_HR_* for the STS and LTS for the combination with HR. Likewise, we use Δ*LTS_RMSSD_* and Δ*STS_RMSSD_* for the combination with RMSSD. We use these adaptiveness measures to examine research questions 3 and 4.

## 4 Results

The Results are organized into three sections. First, we describe HR- and RMSSD-based cardiovascular response types identified with the GSSG method. Second, we examine the extent of HR and RMSSD up- or downregulation and short- and long-term stability during classroom disruptions. Third, we descriptively explore partial correlations between cardiovascular adaptiveness and endocrine hormones. As the endocrine analyses were exploratory and insufficiently powered for smaller or moderate effects, these correlations are reported as effect-size estimates without significance testing.

### 4.1 Identifying types of cardiovascular adaptability

We were able to differentiate all 36 teachers into distinct categories based on their cardiovascular responses. The first category consists of teachers who have a higher HR during classroom disruptions, as shown in **Figure 2a**. Eight of the examined teachers (22.22%) showed this response. We refer to this first cardiovascular response pattern as “maladaptive” as it was characterized by HR upregulation, like in **Figure 2a**. Additionally, most upregulating teachers (six out of eight) show lower HR short-term and long-term stability.

In contrast, 18 teachers (50%) showed the opposite cardiovascular response: during classroom disruptions, they downregulated their HR and had more short-term and long-term stability regarding HR, like in **Figure 2b**. In contrast to the “maladaptive” type, we refer to this type as “stable adaptive” because these teachers adapt to classroom disruptions through HR downregulation with more stability.

The remaining ten teachers (27.78%) also adapt to classroom disruptions to some extent, like the example in **Figure 3**. They also downregulate their HR, like the stable adaptive teachers. However, they do not show increases in STS and LTS. This lack of increasing stability is why we need a further distinction between downregulating teachers through a third type. Since they can adapt to classroom disruptions to some extent, but their HR is unstable during downregulation, we call them “unstable adaptive”. Thus, in summary, 28 of 36 teachers (77.78%) downregulated their HR, but their downregulation process is either unstable or stable.

**Figure 3:**
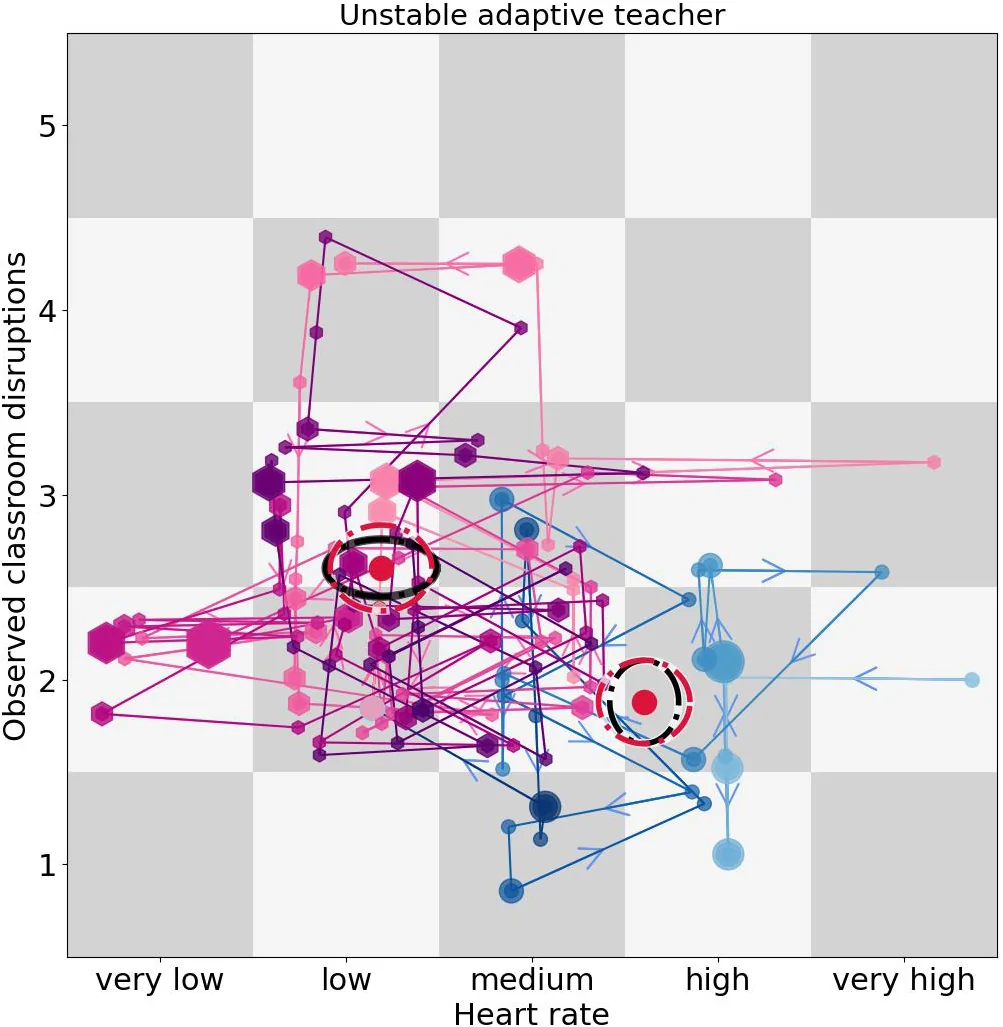
GSSG of a teacher with downregulating HR and decreases in STS and LTS.

Regarding the classifications according to the RMSSD, we found six teachers with the “maladaptive” type, four teachers were “stable adaptive” for RMSSD, and twenty-six were “unstable adaptive”. These four “stable adaptive” teachers were also classified as “stable adaptive” regarding HR. It is important to note that the terms “adaptive” and “maladaptive” are used as operational classifications based on the prespecified cardiovascular response patterns derived from HR regulation and HR short- and long-term stability. They do not represent clinically or prognostically validated categories.

### 4.2 The extent of HR and RMSSD increase, decrease, and stability

Since the GSSG method not only displays the stability of the cardiovascular response but also measures it, we can take a closer look at the cardiovascular response of each type of adaptability.

To validate the categorization of “stable adaptive”, “unstable adaptive”, and “maladaptive”, we compared the change in HR short-term stability with the overall change in the HR through **Figure 4**. The *x*-axis shows the mean value of the HR before classroom disruptions minus the mean value of the HR during the disruptions. Thus, a positive change of $\text{Δ}\bar{HR}$ indicates an overall decrease in HR (downregulation), while a negative change shows an overall increase in HR (upregulation). The *y*-axis shows the subtraction between the HR short-term stabilities. Similar to $\text{Δ}\bar{HR}$, a positive value indicates an increase in HR stability, while a negative value displays a decrease.

**Figure 4:**
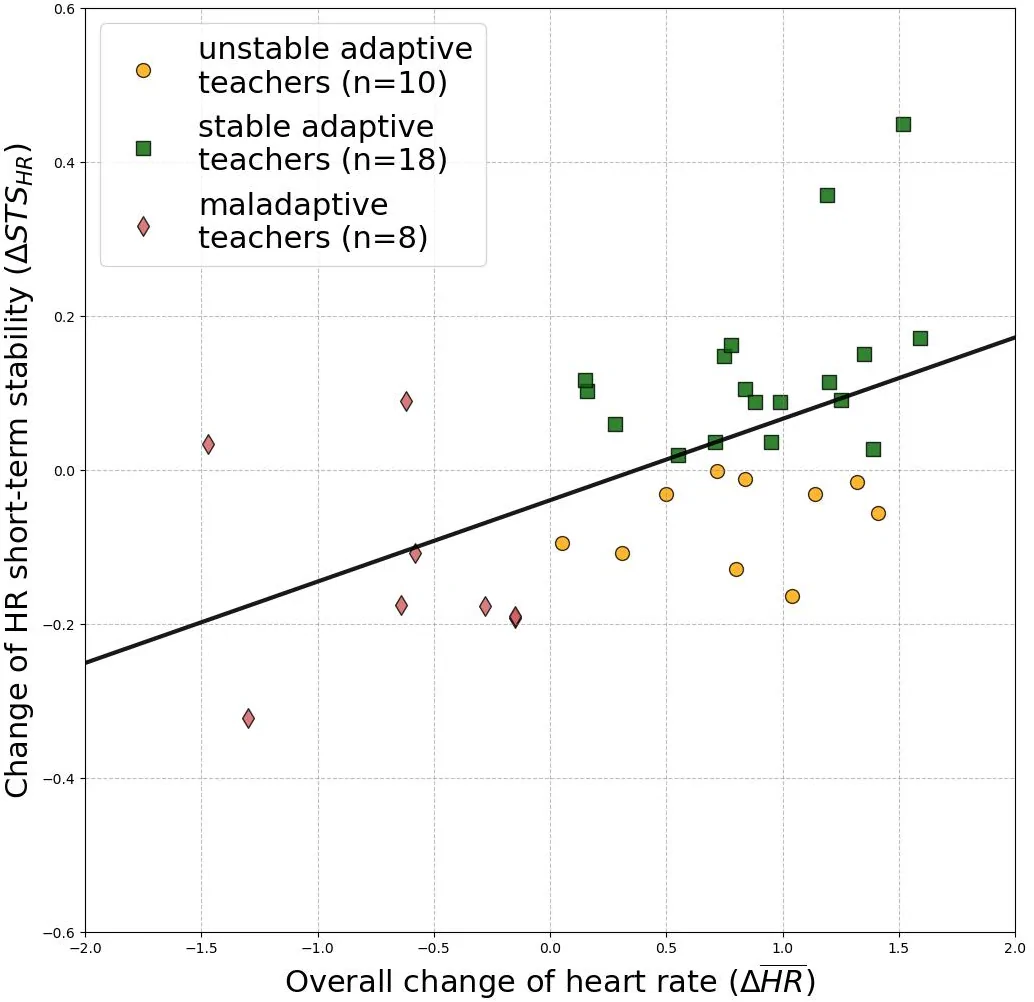
Relations between the change of HR stability (Δ*STS_HR_*) and the overall change in the HR ( $\text{Δ}\bar{HR}$). A positive change in stability indicates an increase in stability during disruptions.

In **Figure 4**, we find three distinctive domains in each quadrant of the coordinate system: one domain where the overall change of the heart rate $\left(\text{Δ}\bar{HR}\right)$ is negative, and two domains with positive $\text{Δ}\bar{HR}$, which are distinguished through a positive change in STS (above 0) and a negative change in STS (below 0). Through this clustering of the coordination system into quadrants, we again find the same three categories of adaptiveness: 1) the teachers of the “maladaptive” type (in red) in the quadrant with negative $\text{Δ}\bar{HR}$, as they increase their HR, 2) the “stable adaptive” teachers with a positive $\text{Δ}\bar{HR}$ and a positive $\text{Δ}STS_{HR}$, as they decrease their HR and increase the stability, and 3) the ”unstable adaptive” teachers with positive $\text{Δ}\bar{HR}$ and negative $\text{Δ}STS_{HR}$, as they also decrease their overall value of HR but decrease their stability. Furthermore, we find a positive linear relation for all types of cardiovascular responses between the overall change of HR and the HR short-term stability $r_{short}=.55$ ( p=.001).

Examining the HR short-term stability before and during classroom disruptions also reveals the same precise distribution of the two adaptive cardiovascular responses in **Figure 5**. Since the “stable adaptive” teachers have higher HR short-term and long-term stability during than before disruptions, they are above the dashed diagonal. In contrast, the “unstable adaptive” teachers and most “maladaptive” teachers (6 out of 8) are located below the dashed line because they exhibit lower HR stability during increased disruptions compared to before. Furthermore, we find an additional effect: the two stability measurements exhibit a correlation for the “adaptive” teachers (*r_stable_* = .97, *p <.*001 and *r_unstable_* = .78, *p <.*001).

**Figure 5:**
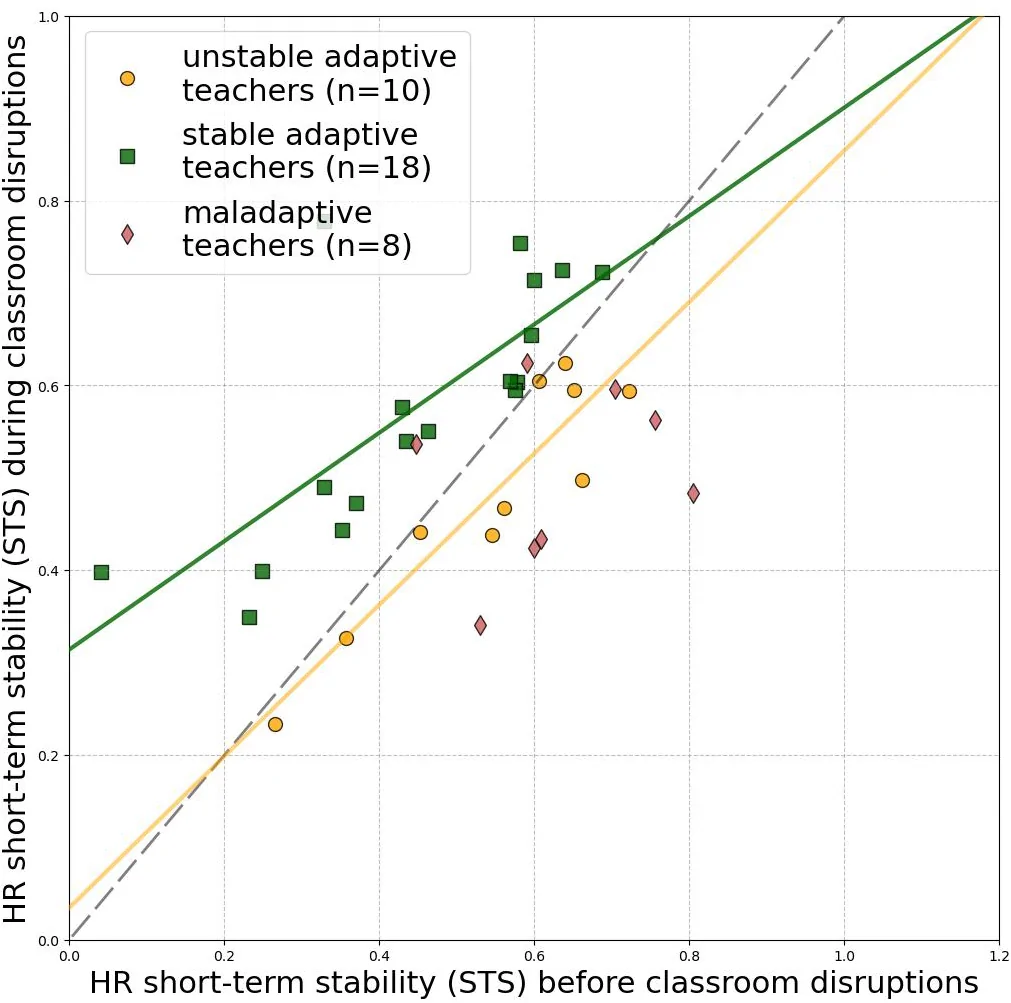
Comparison of the HR short-term stability (STS) before and during classroom disruptions.

Comparing the change in LTS and the overall change in HR (see **Figure 6**) reveals a similar distinction between “maladaptive” and “adaptive” teachers. However, the “unstable” and “stable” clusters are less clear than those for the STS. Furthermore, we find a positive linear relation for all types of cardiovascular responses between the overall change of HR and the HR short-term stability (see **Figure 4**) *r_short_* = .55 (*p* = .001) as well as the HR long-term stability (see **Figure 6**), but to a lesser effect strength of *r_long_* = .37 (*p* = .003).

**Figure 6:**
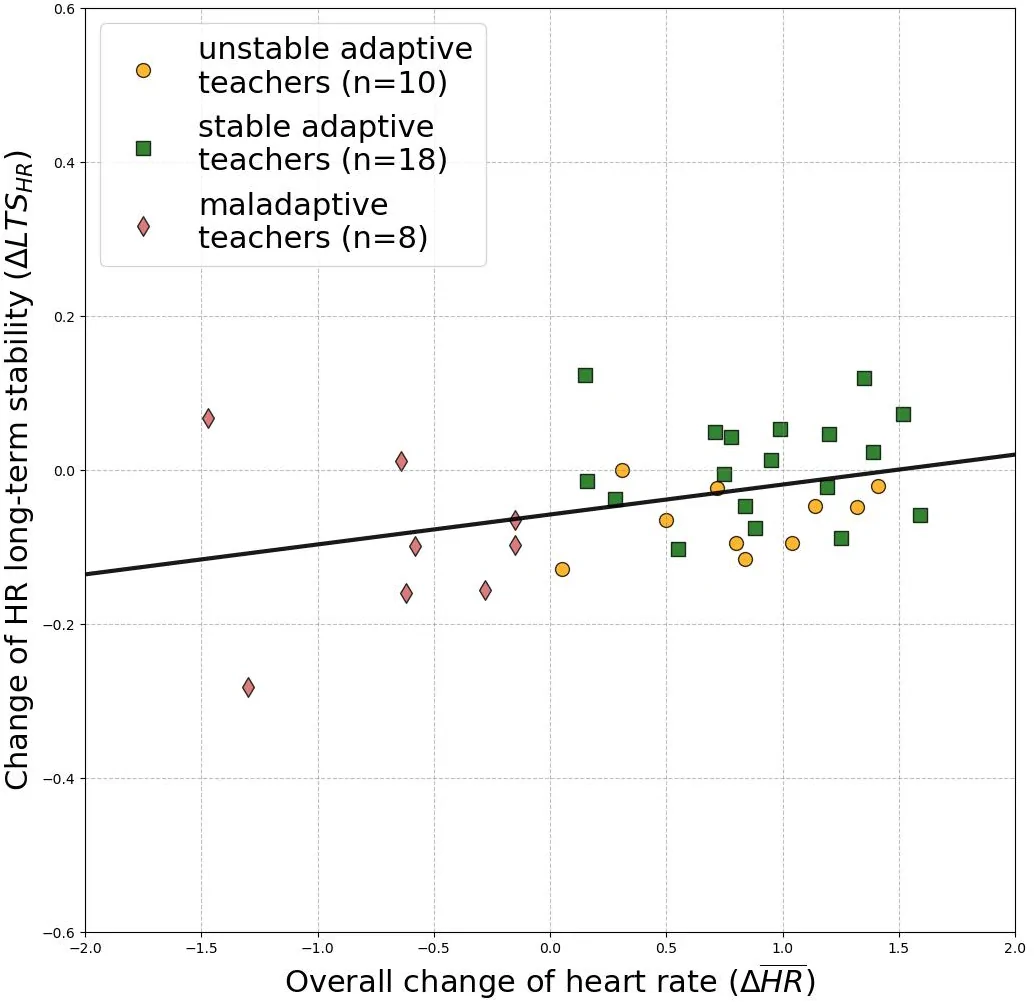
Relations between the change of HR stability (Δ*LTS_HR_*) and the overall change in the HR ( $\text{Δ}\bar{HR}$). A positive change in stability indicates an increase in stability during disruptions.

Not only did we compare the overall change of the HR and the HR’s stability, but we also examined the intensity of classroom disruptions. To calculate the change in intensity of classroom disruptions, we subtracted the mean value of the observed classroom disruptions in the second state from the first state, similar to the overall change in HR. Thus, a negative value indicates an increase in observed classroom disruptions. As **Figure 7** shows, we also find a positive correlation between the change of intensity of observed classroom disruptions and the HR short-term (*r_short_* = .37, *p* = .003) and the HR long-term stability (*r_long_* = .33, *p* = .003).

**Figure 7:**
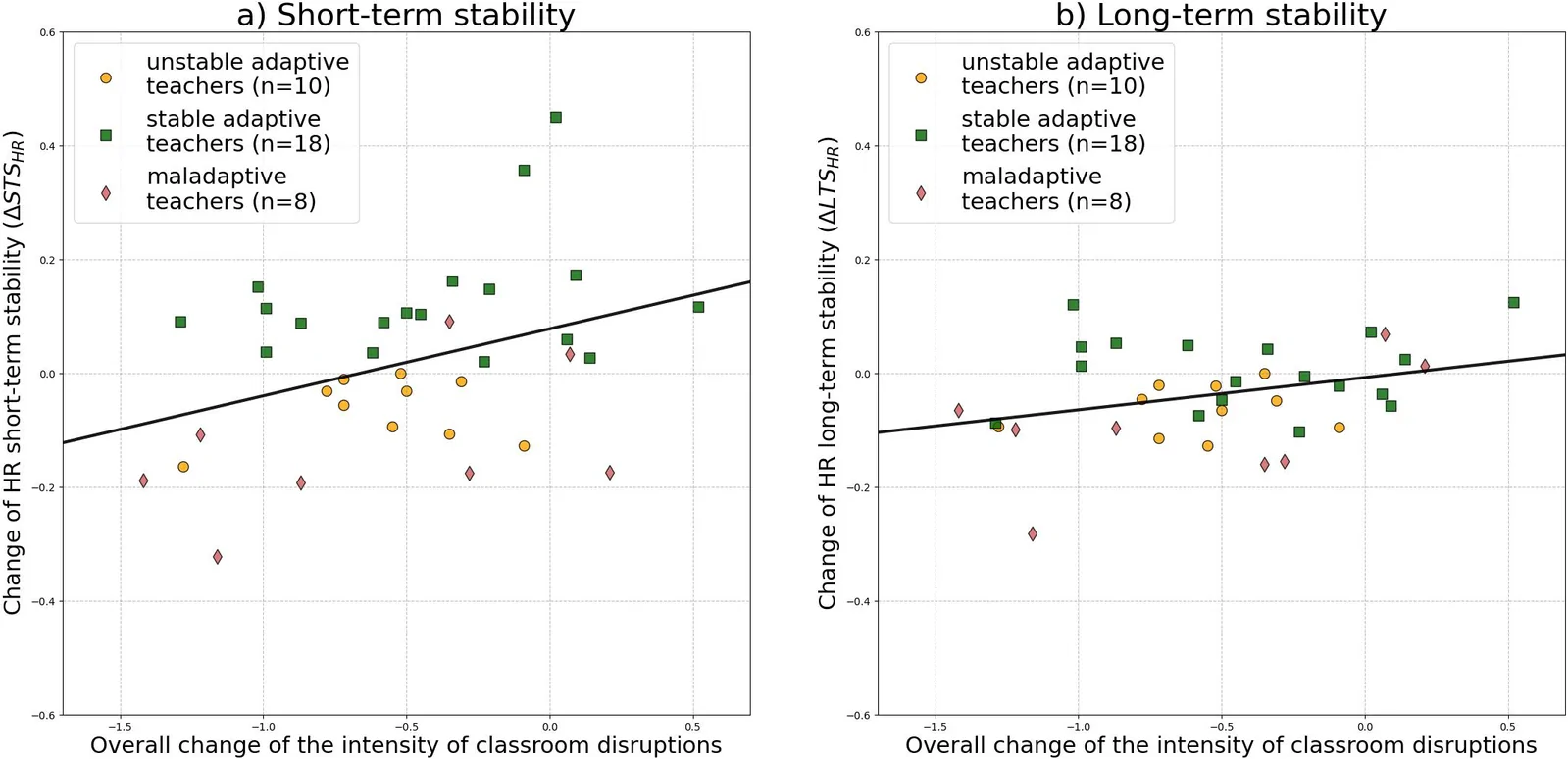
Relations between the overall change of the intensity of classroom disruptions and the change in **(a)** short-term HR stability (Δ*STS_HR_*) on the left side and **(b)** long-term HR stability (Δ*LTS_HR_*) on the right side. A positive change in the intensity of classroom disruptions indicates fewer disruptions, while a positive change in stability indicates increased stability during disruptions.

Since we also measured the RMSSD, we can look at the overall change in RMSSD between the two states, similar to **Figure 4**. Comparing the HR short-term stability with the change in RMSSD, we again find clear distinctions between the three types of cardiovascular responses (see **Figure 8**) and a positive correlation (*r_short_* = .37, *p* = .031). However, we cannot find a correlation between the HR long-term stability and $\text{Δ}\bar{RMSSD}$ (*r_long_* = .26, *p* = .125).

**Figure 8:**
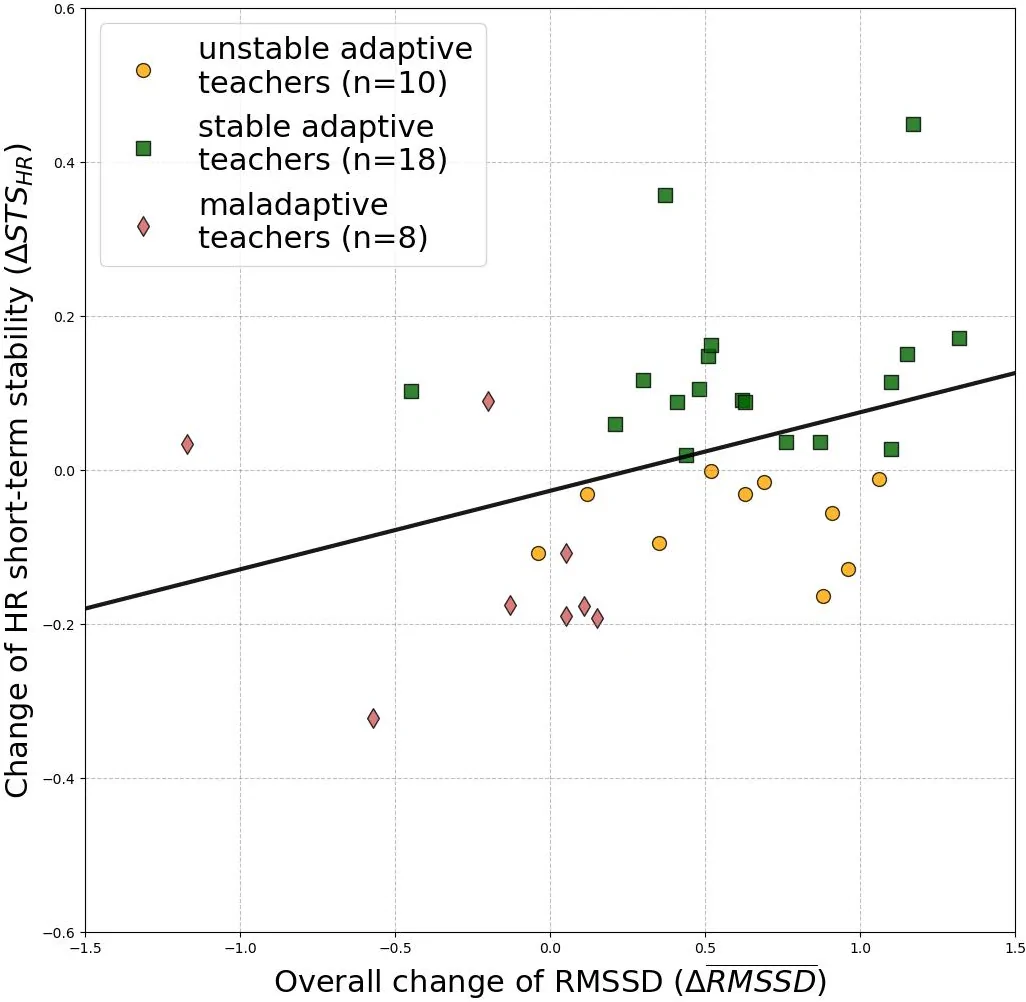
Relations between change of HR short-term stability (Δ*STS_HR_*) and change of the binned and normalized RMSSD $\text{Δ}\bar{RMSSD}$. A positive change in Δ*RMSSD* indicates a higher RMSSD during disruptions. A positive change in Δ*STS_HR_* indicates a higher short-term stability of the teacher’s HR during disruptions.

Looking at the possible correlation between the RMSSD short-term as well as the RMSSD long-term stability and its overall change ( $\text{Δ}\bar{RMSSD}$), we can only find a significant negative correlation for the maladaptive type (*r_long,maladaptive_* = −.77, *p* = .003 and *r_short,maladaptive_* = −.86, *p* = .039). A significant correlation over all three types does not exist (*r_long_* = −.26*, p* = .13; *r_short_* = −.25*, p* = .15).

### 4.3 Correlations between cardiovascular adaptiveness and endocrine hormones

To examine research question 4, we descriptively explored partial correlations between the cardiovascular adaptiveness measures and endocrine hormones (**Table 1**). Higher values in $\text{Δ}\bar{HR}$, Δ*STS_HR_*, and Δ*LTS_HR_* indicate greater cardiovascular adaptiveness during classroom disruptions. As stated above, these analyses were treated as exploratory effect-size estimates and were interpreted in terms of the direction and magnitude of the observed associations, without significance testing. $\text{Δ}\bar{HR}$ showed negative associations with the C/DHEA ratio at *t*_0_ and *t*_1_ (*r_t,_*_0_ = −.52; *r_t,_*_1_ = −.49), when controlling for age, sex, body mass index, and teacher absence. Δ*STS_HR_* showed negative associations with HCO (*r_t,_*_0_ = −.58; *r_t,_*_1_ = −.54) and the sum of HCC and HCO (*r_t,_*_0_ = −.45; *r_t,_*_1_ = −.50) at both measurement points. Δ*LTS_HR_* showed negative associations with the C/DHEA ratio at *t*_0_ and *t*_1_ (*r_t,_*_0_ = −.52; *r_t,_*_1_ = −.53) and a positive association with DHEA at *t*_1_ (*r* = .44). For completeness, we also descriptively explored the partial correlations between the RMSSD adaptiveness measures (**Table 1**) and endocrine hormones. These associations were generally weaker and less consistent than the HR-based associations. The most pronounced RMSSD-based associations were observed between $\text{Δ}\bar{RMSSD}$ and HCC at *t*_1_ (*r* = .40), and the C/DHEA ratio at *t*_0_ and t_1_ (*r_t,_*_0_ = .38; *r_t,_*_1_ = .49).

**Table 1:** Correlations between cardiovascular adaptiveness and endocrine hormones.

Variable	$\text{Δ}\bar{HR}$	$\text{Δ}STS_{HR}$	$\text{Δ}LTS_{HR}$	$\text{Δ}\bar{RMSSD}$	$\text{Δ}STS_{RMSSD}$	$\text{Δ}LTS_{RMSSD}$
HCC $t_{0}$	-.27	-.24	-.25	.25	-.11	-.12
HCC $t_{1}$	-.29	-.35	-.24	.40	-.08	-.06
HCO $t_{0}$	-.18	-.58	-.20	.17	-.22	-.30
HCO $t_{1}$	-.18	-.54	-.24	.26	-.27	-.16
HCCHCOsum $t_{0}$	-.25	-.45	-.25	.23	-.18	-.21
HCCHCOsum $t_{1}$	-.23	-.50	-.26	.32	-.22	-.14
DHEA $t_{0}$	.29	-.06	.30	-.15	-.10	-.33
DHEA $t_{1}$	.34	-.06	.44	-.24	.01	-.09
C/DHEA ratio $t_{0}$	-.52	-.18	-.52	.38	-.04	.18
C/DHEA ratio $t_{1}$	-.49	-.19	-.53	.49	-.06	.04

*n_t_*_0_ = 32, *n_t_*_1_ = 29, *t*_0_ = first measurement, *t*_1_ = one year later, $\text{Δ}\bar{HR}$ = heart rate difference between the two states before and during classroom disruptions, positive values indicate HR downregulation, negative values indicate HR upregulation, $\text{Δ}\bar{RMSSD}$ = the root mean square of distances between successive R peaks difference between the two states before and during classroom disruptions, positive values indicate RMSSD increase, negative values indicate RMSSD decrease, Δ*STS* = short-term stability difference between the two states, Δ*LTS*, long-term stability difference between the two states, HCC, hair cortisol concentration; HCO, hair cortisone concentration; HCCHCOsum, sum of HCC and HCO; DHEA, dehydroepiandrosterone; C/DHEA, hair cortisol - dehydroepiandrosterone ratio; controlled for age, sex, body mass index, and teacher absence during the lesson, no significance testing as purely exploratory, therefore no *p*-values are reported.

## 5 Discussion

In this study, we examined teachers’ HR and HRV (i.e., RMSSD) responses to observed classroom disruptions. Thereby, we took a closer look at HR and RMSSD up- and downregulation, as well as stability, identification of adaptive and maladaptive response types, and associations with endocrine hormones. While previous studies mainly relied on cross-sectional self-reports to assess teacher stress (Aloe et al., 2014b; McGee et al., 2023), we explored the dynamic time development of teachers’ cardiovascular adaptiveness to classroom disruptions using the novel GSSG method (Litzenberger et al., 2025).

Regarding research question 1, we examined how many teachers showed HR and RMSSD downregulation and upregulation in response to observed classroom disruptions, and how stable this regulation was. Results revealed that while most teachers were able to downregulate their HR during classroom disruptions, one quarter of the teachers upregulated their HR. This result is consistent with a study by Junker and colleagues (Junker et al., 2021), who found that although teachers’ HR responses to disruptive-disrespectful student behavior varied, most teachers exhibited a downregulation pattern. Similarly, six out of 36 teachers decreased their RMSSD during classroom disruptions. This distribution indicates a trend that, for most teachers, the vagal brake kicks in (i.e., high cardiac vagal tone, HR decreases, RMSSD increases) in response to a socially stressful classroom situation. A smaller part of teachers, however, reacts with cardiovascular upregulation (i.e., HR increases, RMSSD decreases).

Furthermore, teachers who responded with high cardiac vagal tone showed higher HR short- and long-term stability compared to teachers with a pattern of cardiovascular upregulation. To return to our introduced metaphor, this means that teachers who manage to physiologically downregulate HR and increase RMSSD in response to classroom disruptions (i.e., changing gears in time in front of a hill) tend to also display physiologically more stability during disruptions (i.e., steadily finding the clutch’s friction point).

Regarding research question 2, we attempted to identify more or less adaptive HR and RMSSD response types to observed classroom disruptions. We found three cardiovascular response types: (1) the stable adaptive type, (2) the maladaptive type, and (3) the unstable adaptive type. The stable adaptive type was the largest group of teachers, as they showed decreased HR and heightened HR short- and long-term stability during classroom disruptions. They were able to stably downregulate their physiological arousal in socially challenging situations (i.e., correctly switching gears and steadily finding the clutch’s friction point). This response is stable adaptive, as their physiological reactivity to social stressors is downregulated and they habituate to the stressor (Epel et al., 2018). Our stable adaptive type reflects the ‘habituator profile’ proposed by Hughes et al. (2018), which is expected to lead to optimal health outcomes.

In contrast, the maladaptive type was characterized by HR upregulation and a tendency for lowered HR short- and long-term stability during classroom disruptions. They unstably upregulate their physiological arousal during socially challenging situations, which reflects a pattern of sustained cardiovascular activation (i.e., incorrectly switching gears and unsteadily finding the clutch’s friction point). This response may be considered less adaptive in the present classroom context, as the increased cardiovascular activation could reflect heightened vigilance and may not optimally match the social demands of the classroom situation (Epel et al., 2018). Over time, this pattern can indicate reduced habituation. Although these teachers repeatedly experience classroom disruptions, their cardiovascular responses suggest they may have greater difficulty maintaining effective adaptiveness to this stressor. In this sense, the maladaptive type reflects teachers with habitual patterns of acute physiological overreaction to classroom disruptions. This inflexibility of the cardiovascular system to adapt probably leads to excessive physiological wear and tear (Hughes et al., 2018; Juster et al., 2010). The maladaptive type may correspond to the “persistent reactor” profile described by Hughes et al. (2018), that is, an individual who shows exaggeratedly high cardiovascular reactivity to an initial stressor and continues to respond strongly to subsequent stressors, as predicted by the cardiovascular reactivity hypothesis (Obrist, 1981).

In between was the unstable adaptive type, consisting of teachers who were able to downregulate their HR in response to classroom disruptions, but did this in an unstable way, as their HR short- and long-term stability was decreased. Teachers with this response pattern showed initial HR downregulation, but this downregulation was accompanied by lower HR short- and long-term stability during socially challenging situations (i.e., correctly switching gears but unsteadily finding the clutch’s friction point). These teachers show an adaptive initial response to social stressors, while sustained stable physiological regulation during recurring classroom disruptions appeared less pronounced (Epel et al., 2018; Hughes et al., 2018).

Compared to teachers of the stable adaptive type, those with a maladaptive response type may have a history of greater chronic stress. Prolonged stress may have recursively negatively affected their acute stress reaction, promoting the development of maladaptive response patterns (Epel et al., 2018; Wettstein et al., 2026). Additionally, the maladaptive type is likely to lack recovery and show heightened anticipatory stress prior to the stressor compared to the adaptive type. Moreover, maladaptive response patterns can be harmful to one’s health. A meta-analysis on cardiovascular reactivity to acute mental stress and subsequent cardiovascular disease showed that heightened reactivity and slower recovery from stress predicted poorer cardiovascular outcomes (e.g., hypertension) at the follow-up (Chida and Steptoe, 2010). Thus, the type of cardiovascular reactivity plays a crucial role in the development of cardiovascular diseases (Hughes et al., 2018; Lovallo, 2011; Treiber et al., 2003).

Regarding research question 3, we examined the extent to which teachers’ HR and HRV (i.e., RMSSD) increase, decrease, or remain stable in response to observed classroom disruptions. We found that HR downregulation is positively associated with HR short- and long-term stability during classroom disruptions. This finding suggests that effective adaptability to the first occurrence of classroom disruptions promotes more effective adaptiveness to following, recurring disruptions. However, results revealed that an increase in classroom disruptions was negatively related to HR short- and long-term stability. This result indicates that the more intense the classroom disruptions, the more difficult it is to stabilize the HR, even for teachers of the stable adaptive type. Thus, regardless of the type of cardiovascular response, the more intense classroom disruptions become, the more difficult it is for a teacher to adapt physiologically. Hence, there seems to be a physiological limit to the extent to which a teacher can stabilize their HR. Ultimately, no teacher is ‘immune’ to classroom disruptions and, thus, effective classroom management (Aloe et al., 2014a, b) is crucial.

Further, HR short-term stability was positively associated with the overall change in RMSSD. As elevated RMSSD is an indicator for adaptability to stressors and the ability to regulate stress and arousal (McGee et al., 2023; Thayer et al., 2009), it seems reasonable that it was positively related to teachers’ ability to maintain a stable HR during classroom disruptions. Lastly, we found a negative association between the overall change of RMSSD $\left(\text{Δ}\bar{RMSSD}\right)$ and the RMSSD short- and long-term stability for the teachers of the maladaptive type. However, for both stable and unstable adaptive teachers, this relation was not significant. This disparity in significance suggests that the maladaptive type may be particularly at risk in terms of cardiovascular health. In contrast, this seems irrelevant for the stable and unstable types, as a change in RMSSD was not related to RMSSD stability.

Regarding research question 4, we descriptively explored how cardiovascular adaptiveness measures are associated with endocrine hormones. As noted above, these analyses were treated as exploratory effect-size estimates and interpreted without significance testing. Higher values in $\text{Δ}\bar{HR}$, Δ*STS_HR_*, and Δ*LTS_HR_* indicated greater HR-based cardiovascular adaptiveness to increased classroom disruptions. Overall, the descriptive pattern suggested that greater HR-based cardiovascular adaptiveness tended to be associated with a more favorable endocrine profile.

In particular, Δ*STS_HR_* showed negative associations with HCO and HCCHCOsum at *t*_0_ and *t*_1_. Thus, short-term HR-based cardiovascular adaptiveness to increasing classroom disruptions was negatively associated with HCO and the sum of HCC and HCO. This pattern is broadly consistent with previous studies that have found inverse associations between HCC and HCO and cardiovascular health (Iob and Steptoe, 2019; Kuehl et al., 2015; Stalder et al., 2013). Interestingly, in the study by Stalder et al (Stalder et al., 2013), HCO also showed a stronger association with cardiovascular risk factors than HCC.

Furthermore, $\text{Δ}\bar{HR}$ and Δ*LTS_HR_* showed negative associations with the C/DHEA ratio at *t*_0_ and *t*_1_. Additionally, Δ*LTS_HR_* showed a positive association with DHEA at *t*_1_. Taken together, these two measures of HR-based cardiovascular adaptiveness to classroom disruptions were descriptively associated with higher physiological resilience (i.e., DHEA) and lower physiological dysregulation (i.e., C/DHEA ratio). These results align with previous findings that highlight the protective role of DHEA for the cardiovascular system and the cardiovascular risks associated with physiological dysregulation (Kivimäki and Steptoe, 2018; Mannella et al., 2018; Mannic et al., 2015).

The RMSSD-based associations with endocrine markers were generally weaker and less consistent than the HR-based associations. This pattern may indicate that HR-based short-term and long-term stability can be distinguished from RMSSD-based measures, which reflect HRV.

The concept of metabolic uncoupling may provide a theoretical perspective for generating hypotheses about the observed associations between cardiovascular responses and the endocrine stress markers cortisol and cortisone (Balanos et al., 2010; Carroll et al., 2009). It posits that psychosocial stressors can elicit cardiovascular reactions that are disproportionately strong relative to the energy expenditure required by the situation. In this context, teachers who responded to classroom disruptions with HR upregulation may have exhibited metabolically exaggerated cardiac reactions, as the expected energy expenditure in the classroom is unlikely to account for the magnitude of the observed response. Such a response may be considered less adaptive from a physiological perspective, as a mismatch between heightened physiological activation and actual demands in the classroom could be one possible mechanism linking HR upregulation to less favorable HCC and HCO profiles.

More broadly, previous research has linked cortisol and cortisone levels, as well as HPA-axis dysregulation, to cardiovascular functioning. Elevated HCC has been associated with the incidence of cardiovascular disease (CVD), a leading contributor to the burden of morbidity and mortality across the world (GBD 2017 Causes of Death Collaborators, 2018; Iob and Steptoe, 2019). HCO has also been linked to adverse cardiometabolic markers (e.g., metabolic syndrome; Kuehl et al., 2015; Stalder et al., 2013). In contrast, DHEA might provide protective effects for the cardiovascular system (Mannella et al., 2018; Mannic et al., 2015). As previously suggested, dysregulation of the HPA-axis can be an underlying mechanism of the adverse physiological stress effects on CVD (Kivimäki and Steptoe, 2018). Accordingly, dysregulation in the form of high cortisol and low DHEA might pose a health risk for the cardiovascular system, although the present study cannot establish directional or causal pathways.

Which factors may support more adaptive physiological response patterns to social stressors in the classroom? Threat and challenge appraisal could provide a meaningful intervention area, as appraisals are modifiable. The way teachers think about their bodily responses could improve their physiological and behavioral reactions to social stressors. Both threat and challenge appraisals can activate the ANS. However, threat appraisals induce greater HPA-axis activation, which can be physiologically harmful (e.g., prolonged high cortisol and low DHEA levels; Blascovich, 2008; Jamieson et al., 2018b). For instance, in the study of Jamieson et al. (2012), participants instructed to reappraise their arousal as functional exhibited increased perceptions of available resources, more adaptive cardiovascular stress responses (i.e., increased cardiac efficiency and lower vascular resistance), and decreased threat-related attentional bias. Hence, if teachers appraise classroom disruptions as challenges that can be addressed with effective classroom management, this may support adaptive physiological responses. Such appraisal fosters perceived safety, and teachers are more likely to react via increased vagal tone than sustained cardiovascular upregulation (Porges, 2003).

An essential moderator of whether teachers’ stress responses become adaptive or maladaptive might be stress beliefs (or “stress mindsets”). They reflect individuals’ attitudes toward stress, such as viewing it as harmful to health or productivity, and represent meta-cognitive processes that shape whether stress is interpreted as debilitating or enhancing (Laferton et al., 2023). According to Jamieson et al. (2018a), negative stress beliefs foster threat appraisals and positive stress beliefs promote challenge appraisals. Another promising approach is biofeedback: becoming aware of and controlling unconscious bodily functions. Studies indicate that biofeedback can improve physiological stress indices such as HR, HRV, and cortisol level (Berry et al., 2014; Dillon et al., 2016; Kotozaki et al., 2014).

## 6 Limitations and strengths

First, given the explorative design of the present study, one limitation is the relatively small sample size of healthy, medication-free teachers, which limits the generalizability of the findings. This limitation is particularly relevant for the exploratory endocrine analyses. Given the limited sample size, the correlations between cardiovascular adaptiveness measures and endocrine hormones were reported descriptively as effect-size estimates and interpreted without significance testing. These associations should therefore be interpreted cautiously, require replication in larger samples, and should not be interpreted as evidence for causal, directional, or bidirectional relationships between the ANS and the HPA-axis. Instead, they should be understood as hypothesis-generating associations that may inform future confirmatory studies. Due to the high effort involved in participating in the study, it can be assumed that the participating teachers were more resilient or less stressed than the average teacher. Second, as this was a field study, several factors could not be controlled for. For example, teachers were allowed to choose the day of video-taping and ECG recording, so that teachers may have chosen working days with lower or different stress intensities. Third, due to the high effort necessary for analyzing the video and ECG data, data was assessed only on one working day. Therefore, the results strongly depend on the condition and behavior of teachers and students on the day of data collection. Lastly, individual differences such as psychological resilience, baseline chronic stress, or habitual appraisal tendencies may have influenced teachers’ cardiovascular response patterns.

Nonetheless, this study has several strengths. The combination of behavioral observation, electrophysiological measures, and assessment of endocrine hormones increases the ecological validity of the presented results. The novel GSSG method enables the comparison of two types of stabilities (LTS and STS) of HR and RMSSD. While we found no direct correlation between the LTS and STS of the RMSSD and its overall change during classroom disruptions, we found a clear correlation within each type of teacher adaptiveness for the LTS and STS of the HR, as well as a correlation between HR stability and RMSSD. This type of approach to HR and HRV measures is the first of its kind. Our findings on correlations between LTS and STS of the HR and other endocrine hormones further strengthen the broad applicability of the GSSG method.

Few studies assess the physiological stress response outside of the laboratory. Although much is known about cortisol, associations with HCO, DHEA, and the C/DHEA ratio are less well studied. Thus, this study extends the limited literature on these endocrine hormones in natural settings. The study identified potential adaptive and maladaptive cardiovascular responses in stressful social interactions and explored their relationship to physiological chronic stress and dysregulation. We assessed endocrine hormones in conjunction with potentially sub-clinically relevant measures (HR short- and long-term stability). Thereby, the study contributes to the scarce literature examining associations between cardiovascular regulation and neuroendocrine markers in the context of psychosocial stressors.

## 7 Conclusion

The present study revealed a trend that most teachers react with increased vagal tone (i.e., HR decreases and RMSSD increases) to observed classroom disruptions. A smaller part of teachers react with cardiovascular upregulation (i.e., sustained cardiovascular activation). Furthermore, through the novel GSSG method, three types of cardiovascular adaptiveness could be identified: a maladaptive pattern, a stable adaptive pattern, and an unstable adaptive pattern. The maladaptive pattern was characterized by HR upregulation and a lack of habituation (i.e., low HR stability) during recurring classroom disruptions. The stable adaptive type consists of teachers exhibiting HR downregulation and habituation (i.e., high HR stability) in response to classroom disruptions. The unstable adaptive response pattern also involves HR downregulation in response to classroom disruptions, but lacks effective habituation. The results also indicated that, regardless of the type of cardiovascular response, there is a physiological limit in teachers when adapting to classroom disruptions. More adaptive cardiovascular responses to classroom disruptions may be supported by effective classroom management, challenge-oriented appraisal of social stressors, positive stress beliefs, and biofeedback. Moreover, descriptively, greater cardiovascular adaptiveness tended to be associated with lower HCC, HCO, and C/DHEA ratio values and higher DHEA values longitudinally. These exploratory findings suggest that less adaptive cardiovascular response patterns may be linked to less favorable endocrine profiles such as overproduction or an underproduction of endocrine hormones and physiological dysregulation. Thereby, the present study elucidated potential bidirectional associations between the SNS and the HPA-axis.

## Data Availability

The raw data supporting the conclusions of this article will be made available by the authors, without undue reservation.
